# SKP1 promotes YAP-mediated colorectal cancer stemness via suppressing RASSF1

**DOI:** 10.1186/s12935-020-01683-0

**Published:** 2020-12-03

**Authors:** Cong Tian, Tingyuan Lang, Jiangfeng Qiu, Kun Han, Lei Zhou, Daliu Min, Zhiqi Zhang, Dachuan Qi

**Affiliations:** 1 Department of Medical Oncology, Shanghai University of Medicine & Health Sciences Affiliated Sixth People’s Hospital East Campus, No. 222 Huan Hu Xi San Road, Pudong New Area, Shanghai, 201306 People’s Republic of China; 2 Department of Medical Oncology, Shanghai Jiao Tong University Affiliated Sixth People’s Hospital East Campus, No. 222 Huan Hu Xi San Road, Pudong New Area, Shanghai, 201306 People’s Republic of China; 3 Department of Gynecologic Oncology, Chongqing University Cancer Hospital & Chongqing Cancer Institute & Chongqing Cancer Hospital, Chongqing, 400030 Chongqing People’s Republic of China; 4 Department of Gastrointestinal Surgery, Renji Hospital Shanghai Jiao Tong University School of Medicine, Shanghai, 200127 People’s Republic of China; 5 Singapore Eye Research Institute, The Academia, 20 College Road, Discovery Tower Level 6, Singapore, 169856 Singapore; 6 Department of Ophthalmology, Yong Loo Lin School of Medicine, National University of Singapore, 1E Kent Ridge Road, NUHS, Tower Block Level 7, Singapore, 119228 Singapore; 7 Ophthalmology and Visual Sciences Academic Clinical Research Program, Duke-NUS Medical School, 8 College Road, Singapore, 169867 Singapore; 8 Department of General Surgery, Shanghai University of Medicine & Health Sciences Affiliated Sixth People’s Hospital East Campus, No. 222 Huan Hu Xi San Road, Pudong New Area, Shanghai, 201306 People’s Republic of China; 9 Department of General Surgery, Shanghai Jiao Tong University Affiliated Sixth People’s Hospital, No. 600 Yishan Road, Xuhui District, Shanghai, 200233 People’s Republic of China

**Keywords:** SKP1, Colorectal cancer, YAP, Stemness, RASSF1

## Abstract

**Background:**

Cancer stem cells (CSCs) have been recognized as an important drug target, however, the underlying mechanisms have not been fully understood. SKP1 is a traditional drug target for cancer therapy, while, whether SKP1 promotes colorectal cancer (CRC) stem cells (CRC-SCs) and the underlying mechanisms have remained elusive.

**Methods:**

Human CRC cell lines and primary human CRC cells were used in this study. Gene manipulation was performed by lentivirus system. The mRNA and protein levels of target genes were examined by qRT-PCR and western blot. The sphere-forming and in vitro migration capacities were determined by sphere formation and transwell assay. The self-renewal was determined by limiting dilution assay. The tumorigenicity and metastasis of cancer cells were examined by xenograft model. The promoter activity was examined by luciferase reporter assay. Nuclear run-on and Chromatin immunoprecipitation-PCR (ChIP-PCR) assay were employed to examine the transcription and protein-DNA interaction. Co-immunoprecipitation assay was used to test protein–protein interaction. The relationship between gene expression and survival was analyzed by Kaplan–meier analysis. The correlation between two genes was analyzed by Spearman analysis. Data are represented as mean ± SD and the significance was determined by Student’s *t* test.

**Results:**

SKP1 was upregulated in CRC-SCs and predicted poor prognosis of colon cancer patients. Overexpression of SKP1 promoted the stemness of CRC cells reflected by increased sphere-forming, migration and self-renewal capacities as well as the expression of CSCs markers. In contrast, SKP1 depletion produced the opposite effects. SKP1 strengthened YAP activity and knockdown of YAP abolished the effect of SKP1 on the stemness of CRC cells. SKP1 suppressed RASSF1 at both mRNA and protein level. Overexpression of RASSF1 abolished the effect of SKP1 on YAP activity and CRC stemness.

**Conclusion:**

Our results demonstrated that SKP1 suppresses RASSF1 at both mRNA and protein level, attenuates Hippo signaling, activates YAP, and thereby promoting the stemness of CRC cells.

## Background

Colorectal cancer (CRC) is a predominant cancer which accounts for about ten percent of cancer-related mortality [1, 2]; this disease can be attributed to factors including age, dietary habits, smoking, obesity, etc. [3, 4]. In spite of emerging new treatments, such as laparoscopic surgery, more-aggressive resection, radiotherapy, neoadjuvant and palliative chemotherapies, few changes in long-term survival rate can be observed [5, 6]. Therefore, the improvement of our understanding about the underlying mechanisms is urgently needed.

The cancer stem cells (CSCs) concept, cancer is fueled by a small population of dedicated stem cells, was proposed decades ago [7, 8]; now, it has been clear that CSCs harbor in a certain niche of tumor tissues for many cancers, and accumulating evidences have shown that CSCs contribute to every important progression of cancer development [9–12], making them attractive as drug targets. However, as the mechanisms underlying the maintenance of cancer stemness have not been fully understood, few strategies eradicating CSCs have been developed. So, exploring novel mechanisms and identifying novel drug targets are important to achieve this goal.

SKP1 (S-Phase Kinase Associated Protein 1) is traditionally known as a component of SCF (SKP1/Cullin-1/F-box) complexes, which are composed of SKP1, cullin 1 and one member of the F-box family proteins, SKP2, for instance [13, 14]. SCF complexes play essential roles in cell cycle progression and organ development by regulating ubiquitination of specific protein substrates for degradation by the proteasome [15, 16]. Recently, evidences have shown that SCF complexes also play crucial roles in cancer and cancer stemness maintenance [17, 18]. For example, ubiquitin ligase subunits, SKP2, a member of F-box family proteins, and CKS1, an important adaptor, promote degradation of cell cycle regulators, such as P21, RASSF1A, and FOXO1, and this mechanism contribute to hepatocellular carcinoma progression [19]. Moreover, SKP2 targets G1/S cyclin-dependent kinase inhibitor (p27) and Akt to induce cell-cycle, glycolysis, and tumorigenesis [20, 21]; knockdown and pharmacological inhibition of Skp2 inhibits ALDH + prostate CSCs [22, 23]. These observations indicate that the components of SCF complex, such as SKP1, SKP2, as well as SKP1-SKP2 interaction, are important drug targets [24]. However, the relationship between SKP1 and CSCs, especially CRC stem cells (CRC-SCs) is not fully understood, and whether SKP1 regulates oncogene or tumor suppressors by SCF complex-independent manner is rarely studied.

Hippo/YAP signaling pathway has been recognized as a linchpin in cancer therapy; dysregulation of core components (MST1/2, LATS1/2, YAP, etc.) associates with initiation, migration, invasion as well as therapeutic resistance of various types of cancer [25]. In Hippo singling, MST1/2 and LATS1/2 constitute a kinase cascade which phosphorylates YAP and inhibits YAP-mediated transcription of target genes by promoting YAP degradation [26]. So far, several regulators of Hippo signaling have been found, including RASSF1, RASSF6, Ajub LIM proteins, PP2A, etc. [25]. There are two potential links between SCF complex and YAP: phosphorylation of YAP recruits the SCFβ-TRCP E3 ubiquitin ligase, which leads to YAP ubiquitination and degradation [27]; SKP2 regulates RASSF1A [19, 28], a YAP negative regulator, which activates MST1/2 by inducing their autophosphorylation [29]. However, the direct experimental evidences of SCF complex-RASSF1A-YAP axis and its role in cancer stemness have remained elusive. Furthermore, as mentioned above, the molecular mechanisms underlying SKP1 are not fully understood.

In this study, we reported that SKP1 suppresses RASSF1 at both mRNA and protein level, attenuates Hippo signaling, activates YAP, and thereby promoting the stemness of CRCs.

## Materials and methods

### Bioinformatic analysis

The gene expression data were downloaded from R2 platform (http://r2.amc.nl) (Additional file 1: sheet 1), the relationship between gene expression and survival was analyzed by Kaplan–meier analysis. The correlation between two genes was analyzed by Spearman analysis. A significant association is indicated by *p* < 0.05.

### Cells culture

Human CRC cell lines (HCT-116 and HT-29) were purchased from the American Type Culture Collection (ATCC, Rockville, MD, USA) and were grown in RPMI 1640 (Thermo Fisher, Waltham, MA, USA) with 10% heat-inactivated fetal bovine serum (FBS, Thermo Fisher, Waltham, MA, USA), 100 units ml^−1^ penicillin (Thermo Fisher, Waltham, MA, USA), and 100 μg ml^−1^ streptomycin (Thermo Fisher, Waltham, MA, USA), in 5% CO_2_ incubator at 37  °C. HEK 293 T cell line was purchased from Clontech Laboratories Inc (Mountain View, CA, USA) and was cultured in RPMI 1640 with 10% heat-inactivated FBS.

For primary culture, the CRC tissues from different areas of the tumor were dissected and was immediately washed with Hank’s balanced salt solution to remove the blood and contaminant. The fat and necrotic tissues were subsequently removed by sterile forceps. The tissues were then minced into pieces of 1 mm^3^ and maintained in serum-free RPMI1640 culture medium supplemented with 2% B-27 supplement (Invitrogen, Thermo Fisher, Waltham, MA, USA), 20 ng ml^−1^ FGF2 (Thermo Fisher, Waltham, MA, USA) and 20 ng ml^−1^ EGF (Thermo Fisher, Waltham, MA, USA). Digestion was performed by adding the collagenase (40 U ml^−1^) (Thermo Fisher, Waltham, MA, USA) to the medium and then the dissociated tissues were passed through cell strainer filter. Erythrocytes were removed by BD Pharm lyse lysing buffer (BD Falcon, Franklin Lakes, NJ, USA). The resulting cells were washed by centrifuge and placed into cell culture dish.

### Clinical samples

All tissues were obtained from Shanghai Jiao Tong University Affiliated Sixth People’s Hospital East Campus, which was approved by ethics committee of Shanghai Jiao Tong University Affiliated Sixth People’s Hospital East Campus. The written informed consent was obtained from each patient. All procedures were conducted in accordance with the Declaration of Helsinki. For correlation analysis, the tumor samples from thirty patients were collected and the mRNA levels of the genes were measured by qRT-PCR (Additional file 1: sheet 2). The data from all these thirty samples were used for each analysis.

### Antibodies, primers and reagents

The antibodies and primers used in the study were listed in Additional file 2. All other reagents were obtained from Sigma-Aldrich (St. Louis, MO, USA).

### Sphere-formation assay

The cells were plated in 6-well plate (Ultra-low attachment) at the density of 7000 cells per well and incubated in stem cell medium (serum free RPMI 1640 medium containing 10 ng ml^−1^ FGF, 10 ng ml^−1^ EGF, 1 × N2 supplement, 100 units ml^−1^ penicillin, and 100 μg ml^−1^ streptomycin). For SKP1-knockdown cells, the spheres were observed after 15–25 days. For SKP1-overexpressing cells, the spheres were observed after 7–15 days. The spheres were observed when the biggest sphere reached a diameter of 100 μm. For sphere passage, the spheres were collected by centrifugation and dissociated with trypsin–EDTA. The cells were then washed and re-suspended in serum-free medium. The spheres should be passaged before they reached a diameter of 100 μm.

### Tumorigenesis and metastasis

Human CRC cell line HCT-116 was transfected with SKP1-overexpressing lentivirus vector or control vector and the stable cell lines were established. Then, 2 × 10^6^ cells in serum-free medium with an equal volume of Matrigel (Thermo Fisher, Waltham, MA, USA) were injected into flank of 8 to 12-week-old female nude mice. Tumors were measured by tumor volume (mm^3^). For lung metastasis examination, 5 × 10^6^ cells in 100 µl PBS were injected via the tail vein (t.v.). After 20 days, mice lungs were fixed and embedded in OCT, followed by H&E staining. The number and diameter of metastatic tumors in lung were recorded under microscope. All the procedures were approved by Institutional Animal Care and Use Committee in Shanghai Jiao Tong University Affiliated Sixth People’s Hospital East Campus (Shanghai, China).

### Genetic manipulation

For overexpression, the coding sequence regions of SKP1 and RASSF1 were cloned into pCDH-CMV-MCS-EF1-Puro lentivirus plasmid, respectively. For knockdown, the pLKO.1 lentivirus particle containing shRNAs against SKP1 (TRCN0000284791, TRCN0000272541) and RASSF1 (TRCN0000077854, TRCN0000077856) were purchased from Sigma-Aldrich Merck (St. Louis, MO, USA). HEK 293T cells were cultured and co-transfected with reconstructed plasmids, VSV-G (envelop plasmid) and delta R8.2 (packaging plasmid) followed by 7-15 days culture. The culture media containing lentivirus particles were then harvested followed by concentration using a 0.45 μm filter and Lenti-X Concentrator (Clontech, Mountain View, CA, USA). The target cells were infected with the lentivirus and selected by puromycin. The stable cell lines were verified by western blot.

### Luciferase reporter assay

For luciferase reporter assay, the promoter region of RASSF1 was cloned into PGL4 luciferase reporter vectors (Promega, WI, USA). A dual-luciferase reporter assay (Promega) was used to measure RASSF1 promoter activity according to the manufacturer’s instructions. Briefly, 3 days before measuring luciferase activity, pGL4-RASSF1 promoter or PGL4 firefly luciferase promoter reporter were transfected into the cells by using Lipofectamine 3000. Additionally, cells were transfected with pRL-CMV, which encodes a Renilla luciferase for cell number normalization. Firefly/Renilla luciferase activity was measured with a luminometer.

### Quantitative real-time reverse-transcription PCR

Total RNA isolation was performed by RNAzol RT reagent (Molecular Research Center, Cincinnati, OH, USA). SuperScript III Platinum SYBR Green One-Step qRT-PCR Kit (Thermo Fisher, Waltham, MA, USA) was used for quantitative reverse transcriptase PCR (qRT-PCR) and GAPDH was used as internal control.

### Western blot

Sodium dodecyl sulfate–polyacrylamide gel electrophoresis (SDS-PAGE) was used for sample separation. Immobilon-P membranes (Millipore-Sigma, St. Louis, MO, USA) servers as a carrier for blotting. The protein samples were first incubated with the primary antibody at 4 °C overnight followed by 2-4 h incubation with related secondary antibody conjugated with horseradish peroxidase (HRP). The signals were produced by Clarity™ Western ECL Substrate (BioRad, Hercules, CA, USA).

### Co-immunoprecipitation

Co-immunoprecipitation was conducted by antibodies as indicated in the figures. Briefly, proteins (about 800 μg) were incubated with indicated antibodies at 4 °C for overnight. Protein A or G beads (Santa Cruz Biotechnology, Santa Cruz, CA, USA) were then added and was incubated at 4 °C for additional 2 h. Beads were washed three times. Bound proteins were detected by western blot with antibodies as indicated in the figures.

### Transwell assay

The transwell assay was performed in a culture insert with permeable membrane (Thermo Fisher, Waltham, MA, USA) according to the manual. Briefly, 2 × 10^3^ cells were placed on the upper layer of the culture insert containing serum-free culture media and the complete media were added in the culture well (Thermo Fisher, Waltham, MA, USA) followed by 18 h culture. The migrated cells were then stained and counted under a phase-contract microscope.

### Limiting dilution assay (LDA)

The cells were cultured in 6 cm dish at the density of 1 × 10^6^ cells per dish. When the cells were reached 80% confluence, the cells were collected and resuspended in ultra-low attachment 96 well plate at the density of 10, 5, 1 cells per well and cultured in serum-free medium. The spheres were observed after 20 days culture. ELDA software was used to determine the frequency of sphere-forming cells.

### Nuclear run-on assay

Click-iT™ Nascent RNA Capture Kit (C10365, Thermo Fisher, Waltham, MA, USA) was used for nuclear run-on assay according to the manual. Briefly, the cells were incubated with culture medium containing 0.5 mM 5-ethynyl uridine (EU) for 1 h. The RNA was then isolated and EU-labled RNA was biotinylated biotin azide (10 ug RNA: 1 mM Biotin Azide), which was subsequentially bound to streptavidin-coupled magnetic beads. The purified EU-labeled RNA was used for cDNA synthesis. The expression of RASSF1 was determined by qRT-PCR.

### Chromatin immunoprecipitation (ChIP) assay

MAGnify™ Chromatin IP System (Thermo Fisher Scientific) was employed for ChIP assay according to the manual. Briefly, the cells were crosslinked with 1% formaldehyde (room temperature, 10 min), followed by incubation with 0.125 M glycine for 5 min. The cells were then collected and incubated with lysis buffer containing proteinase inhibitor (1 h, 4 °C). The lysis was then sonicated to produce 200-500 base pair DNA fragments. After centrifugation (20,000 × g, 10 min), the supernatant was collected. Chromatin samples were then diluted in dilution buffer containing protease inhibitors cocktail, followed by incubation with SKP1 antibody-Dynabeads protein A/G complex (18 h, 4 °C). After wash with IP buffer 1 and 2, the beads were separated and incubated with cross-linking buffer containing proteinase K (55 °C, 15 min), followed by another incubation (65 °C, 30 min). DNA samples were purified by DNA purification magnetic beads. The RASSF1 promoter linked with SKP1 was analyzed by qRT-PCR.

### Cellular fractionation

Protein fractions were prepared by Subcellular Protein Fractionation kit (Thermo Fisher, Waltham, MA, USA) according to the manufacturer’s manual. Briefly, the dissociated cells were first lysed by cytoplasmic extraction buffer (CEB) at 4 °C for 10 min, followed by 5 min centrifugation (500 g). The supernatant including cytoplasmic proteins was transferred into a new tube for subsequent experiments. The rest pellet was incubated with membrane extraction buffer (MEB) at 4 °C for 10 min, followed by centrifugation for 5 min (3000*g*). the nuclear proteins were extracted by incubation of pellet with IB sample buffer and boiled for 5 min. Lamin B1 was used as nucleus internal reference and β-actin was used as cytoplasmic internal reference.

### Statistics

All experiments were performed at least three replicates. Data are represented as mean ± SD and the significance was determined by Student’s t-test.

## Results

### SKP1 is associated with the stemness of CRC cells

**Fig. 1:**
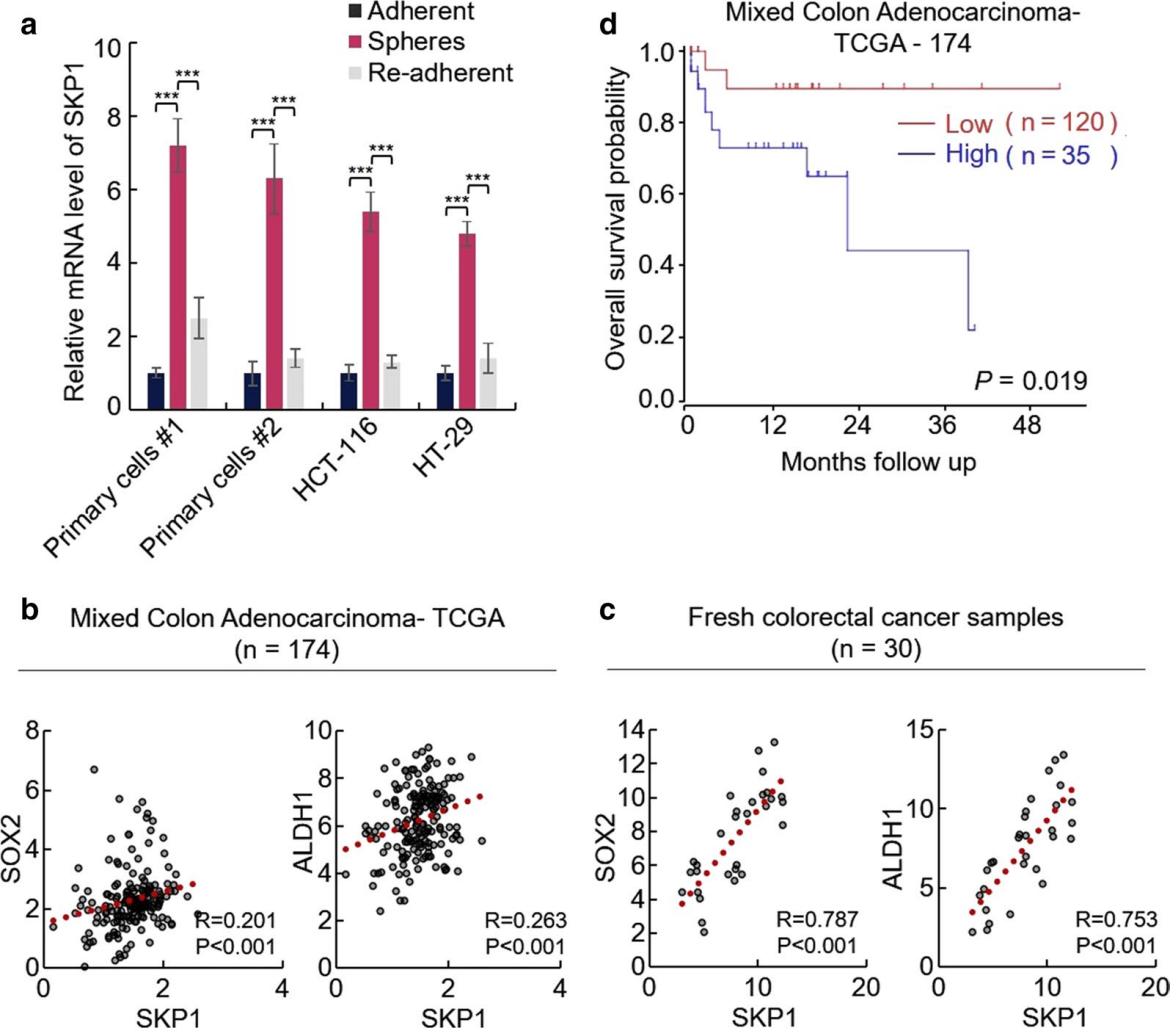
SKP1 is associated with colorectal cancer stemness. **a** The mRNA level of SKP1 is upregulated in CRC spheres, compared with adherent and re-adherent cells. **b**, **c** The expression of SKP1 is positively correlated with cancer stem cell markers. The TCGA dataset was downloaded from R2 platform (**b**) and the mRNA levels of genes in tumor samples from 30 CRC patients were determined by qRT-PCR (**c**). Spearman analysis was performed for correlation analysis. **d** High expression of SKP1 is associated with poor prognosis of colon cancer patients. The TCGA dataset was downloaded from R2 platform. Kaplan–meier analysis was used for analysis of the relationship between SKP1 expression and survival of colon cancer patients. Student’s t-test (***p < 0.001)

CSCs possess the ability to form three-dimensional spheres in suspension culture condition [7–9]. To investigate whether SKP1 is associated with the stemness of CRC-SCs, we first examined the relative mRNA expression level of SKP1 in adherent, suspension cultured sphere and re-adherent CRC cells. As shown in Fig. 1a, a significant increase in mRNA level of SKP1 was observed in sphere cells, indicating the potential role of SKP1 in CRC-SCs.

CRC-SCs are characterized by cell surface markers related to self-renewal and universal stem cell markers, such as ALDH1A1 and SOX2 [7–10]. We next examined the association between SKP1 and CRC-SCs markers in tumor samples. The colon cancer TCGA data were downloaded from R2 platform (http://r2.amc.nl) and the Spearman analysis showed that the expression of SKP1 is positively correlated with SOX2 and ALDH1A1 in CRC samples (Fig. 1b). To confirm this result, we prepared RNA samples from fresh tumor tissues of 30 CRC patients and a similar result was observed (Fig. 1c), which demonstrated the positive correlation between SKP1 and CRC-SCs markers.

The aberrant upregulation of cancer stemness driver gene is commonly associated with poor prognosis [7–9]. We thus investigated the relationship between the expression of SKP1 and the prognosis of patients with TCGA data. As expected, high expression of SKP1 is associated with poor prognosis of colon cancer patients (Fig. 1d). Taken together, these results suggested that SKP1 may play a significant role in stemness maintenance of CRC stemness.

### Ectopic expression of SKP1 promotes the stemness of CRC cells

**Fig. 2:**
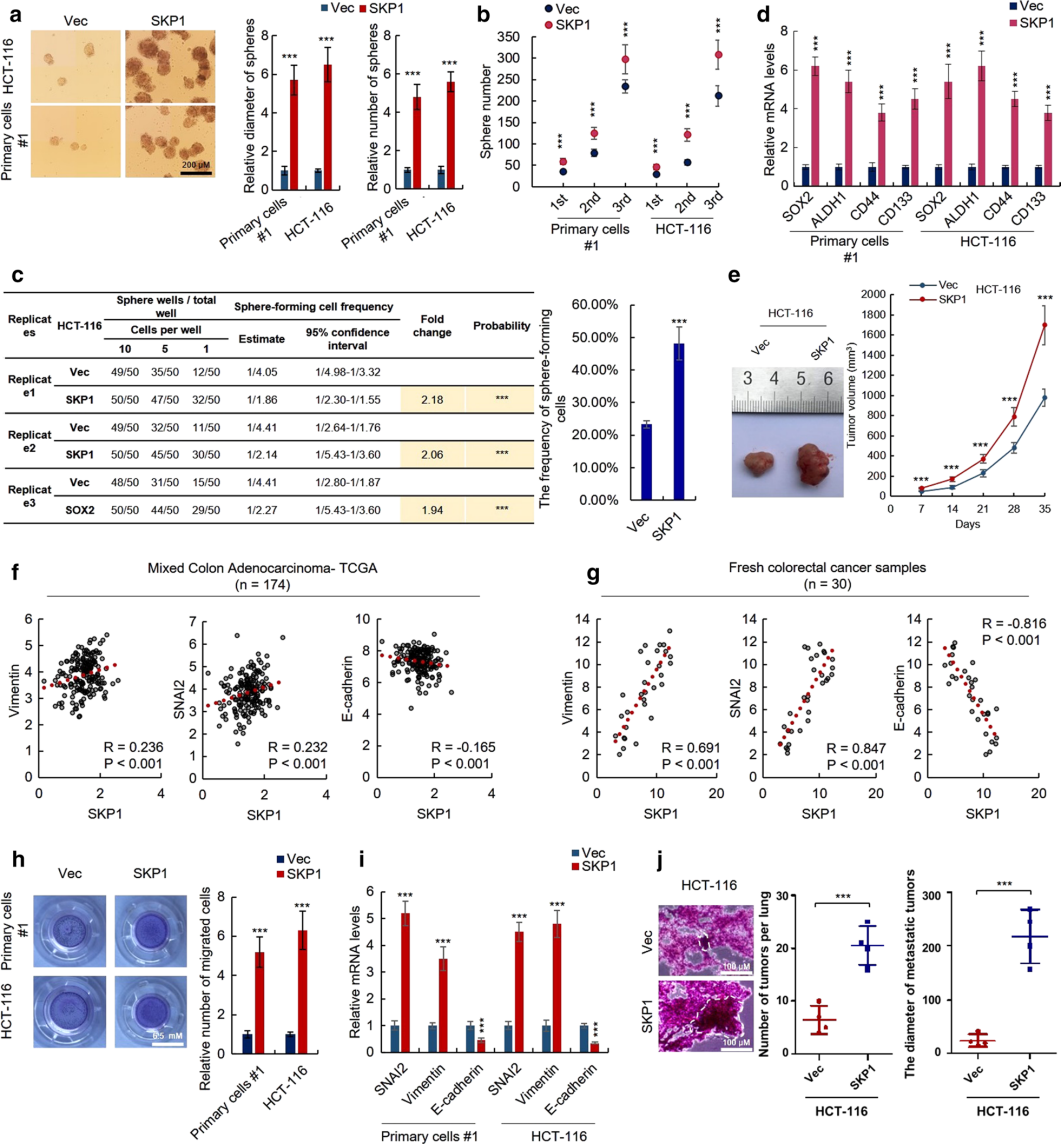
Ectopic expression of SKP1 promotes the stemness and EMT of colorectal cancer cells. **a**, **b** SKP1 promotes the sphere-forming activity of HCT-116 and #1 primary colorectal cancer cells. **a** The sphere-forming assay was performed to determine the sphere-forming activity of HCT-116 and #1 primary colorectal cancer cells transfected with SKP1 or control vectors. **b** HCT-116 and #1 primary colorectal cancer cells with SKP1 overexpression exhibited enhanced sphere-forming capacity on serial passage. **c** SKP1 promotes the frequency of sphere-forming cells in HCT-116 cells. The sphere-forming frequency of HCT-116 cells transfected with SKP1 or control vectors was determined by limiting dilution assay. **d** SKP1 upregulates stem cell markers of HCT-116 and #1 primary colorectal cancer cells. The mRNA levels of indicated genes in indicated cells were determined by qRT-PCR. **e** SKP1 enhances tumorigenic capacity of HCT-116 cells. The tumorigenicity of indicated cells were examined by xenograft model. **f**, **g** The correlation between SKP1 and EMT markers in TCGA (**f**) and 30 fresh tumor samples of CRC patients (**g**) was analyzed by Spearman correlation analysis. **h** SPK1 promotes the migration of colorectal cancer cells. The migration of HCT-116 and #1 primary cells transfected with SKP1 or control vectors was determined by transwell assay. **i** The mRNA levels of indicated EMT markers in SKP1-overexpressing and control HCT-116 and #1 primary colorectal cancer cells were determined by qRT-PCR assay. **j** SKP1 promotes the metastasis of colorectal cancer cells. The number and diameter of lung metastatic tumors were recorded. Student’s t-test (***p < 0.001)

To confirm the association between SKP1 and CRC stemness, we established SKP1-overexpressing HCT-116 and #1 primary CRC cells by lentivirus delivery system (Additional file 2: Figure S1) and examined the properties related to CRC-SCs, including the capacity of self-renewal, expression of related markers as well as tumorigenesis. As expected, we found that overexpression of SKP1 significantly enhanced the sphere-forming capacity of both HCT-116 and #1 primary cells, reflected by sphere diameter and number (Fig. 2a). In addition, the CRC cells with SKP1 overexpression exhibited enhanced sphere-forming capacity on serial passage (Fig. 2b). Moreover, overexpression of SKP1 significantly increased the frequency of sphere-forming cells of HCT-116 cells (Fig. 2c). Furthermore, SKP1 overexpression significantly upregulated the expression of CRC-SCs markers (SOX2, ALDH1, CD44 and CD133) in HCT-116 and #1 primary cells (Fig. 2d). SKP1-overexpressing HCT-116 cells also exhibited enhanced tumorigenic capacity in vivo (Fig. 2e). Taken together, these results confirmed the stimulatory role of SKP1 in stemness maintenance of CRC cells.

### Ectopic expression of SKP1 promotes Epithelial-Mesenchymal transition of CRC cells

Cancer cells often undergo Epithelial-Mesenchymal transition (EMT) to acquire the stemness [10], we next examined whether SKP1 promotes EMT of CRC cells. As shown in Fig. 2f, Spearman correlation analysis with TCGA data showed that the expression of SKP1 is positively correlated with mesenchymal markers (Vimentin, SNAI2) and negatively correlated with epithelial marker (E-cadherin). This result was further confirmed by fresh samples of 30 CRC patients (Fig. 2g). In addition, the in vitro migration capacity of SKP1-overexpressing cells is significantly enhanced by SKP1 overexpression revealed by transwell assay (Fig. 2h). Moreover, the mRNA levels of mesenchymal markers (Vimentin and SNAI2) were significantly upregulated in SKP1-overexpressing cells, while, the mRNA level of epithelial marker (E-cadherin) was significantly downregulated as identified by qRT-PCR (Fig. 2i). Furthermore, the SKP1-overexpressing HCT-116 cells showed enhanced metastatic capacity in vivo (Fig. 2j). These results demonstrated that SKP1 promotes the EMT of CRC cells.

### Knockdown of SKP1 impairs the stemness and EMT of CRC cells

**Fig. 3:**
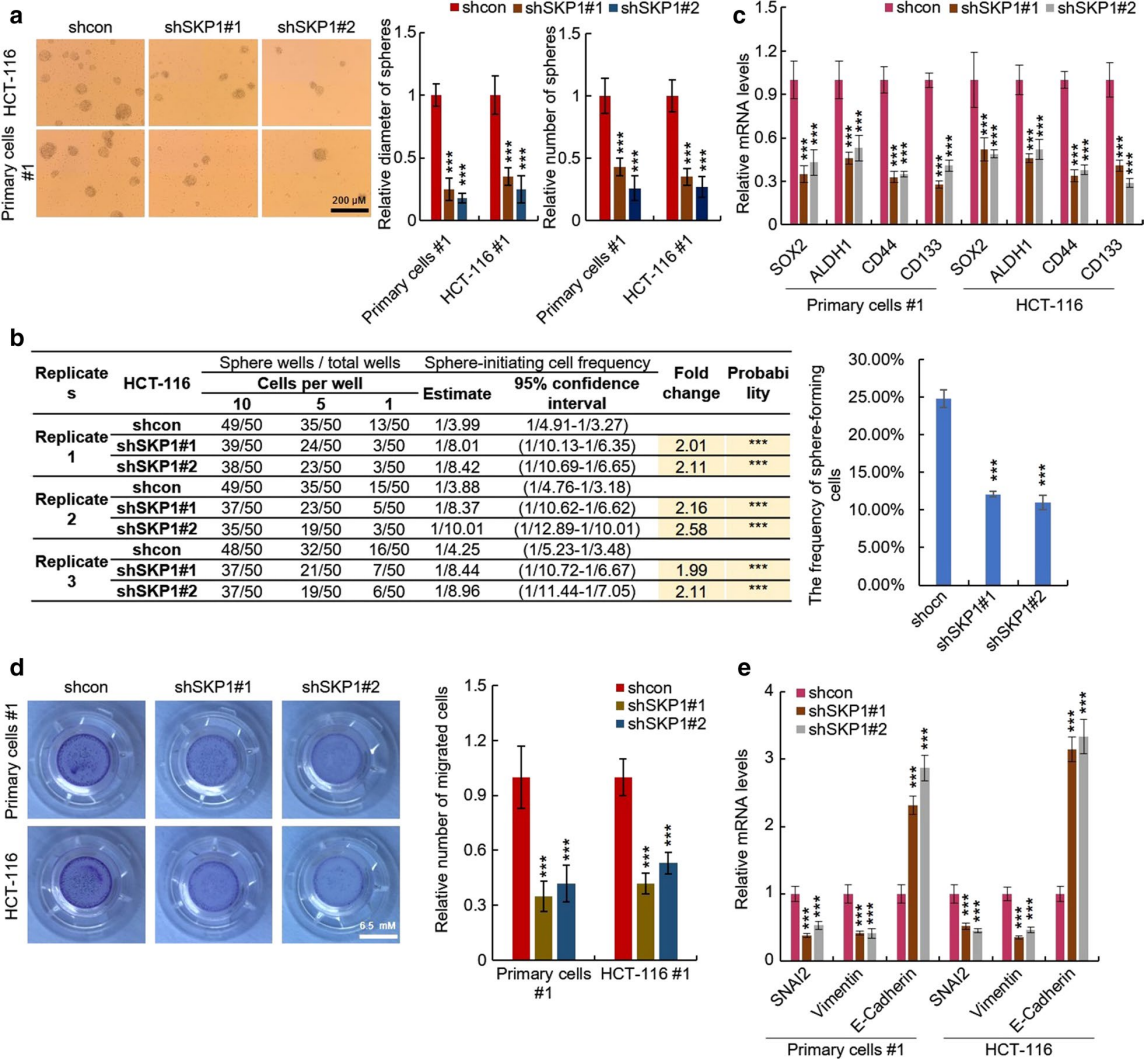
Knockdown of SKP1 inhibits the stemness and EMT of colorectal cancer cells. **a**–**c** Knockdown of SKP1 inhibits the stemness of colorectal cancer cells. The sphere-forming capacity (**a**), frequency of sphere-forming cells (**b**), and expression of cancer stem cell markers (**c**) of SKP1-knockdown and control HCT-116 and #1 primary colorectal cancer cells were determined by sphere formation (**a**), limiting dilution assay (**b**), and qRT-PCR (**c**), respectively. Knockdown of SKP1 inhibits EMT of colorectal cancer cells. The migration (**d**) and expression of EMT markers (**e**) of SKP1-knockdown and control HCT-116 and #1 primary colorectal cancer cells were determined by tranwell (**d**) and qRT-PCR assay (**e**), respectively. Student’s t-test (***p < 0.001)

To further confirm the necessary role of SKP1 in stemness maintenance of CRC cells, the SKP1 was knocked down in HCT-116 and #1 primary colorectal cancer cells (Additional file 2: Figure S2). As expected, SKP1 depletion impaired the stemness of CRC cells reflected by reduced sphere-forming capacity (Fig. 3a), the frequency of sphere-forming cells (Fig. 3b), and the mRNA levels of CRC-SCs markers (SOX2, ALDH1, CD44 and CD133) (Fig. 3c). Furthermore, depletion of SKP1 attenuated EMT of CRC cells reflected by reduced migration capacity (Fig. 3d), mRNA levels of mesenchymal markers (Vimentin and SNAL2) (Fig. 3e) and enhanced epithelial marker (E-cadherin) (Fig. 3e). These results thus demonstrated that SKP1 is necessary for maintaining the stemness of CRC cells. In summary, above results demonstrated that SKP1 promotes the stemness of CRC cells.

### SKP1 activates Hippo/YAP signaling pathway in CRC cells

**Fig. 4:**
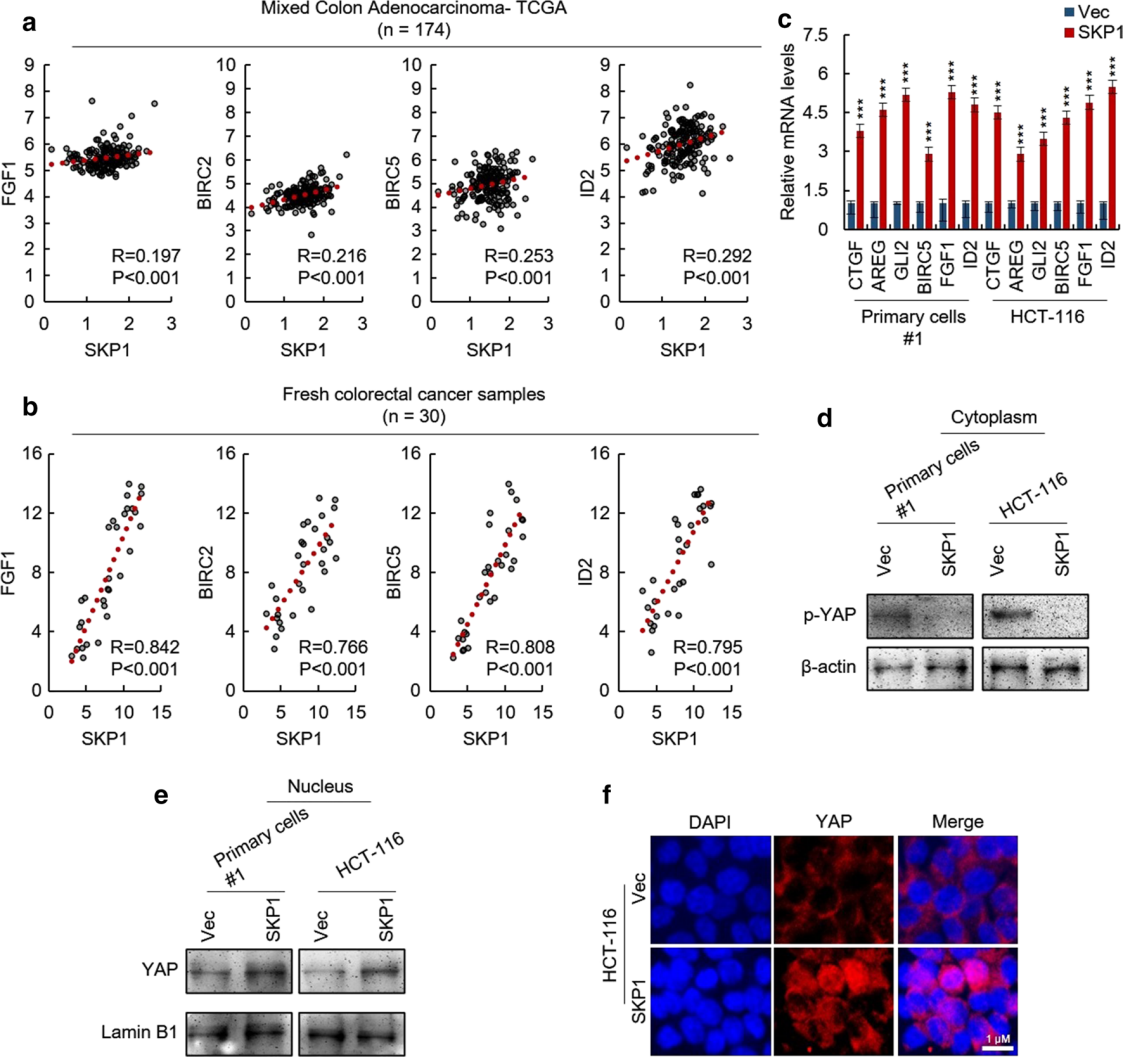
SKP1 positively regulates Hippo/YAP signaling pathway in colorectal cancer cells. **a** The expression of SKP1 is positively correlated with YAP target genes as analyzed by spearman analysis with TCGA datasets. The TCGA dataset was downloaded from R2 platform. Spearman analysis was employed for investigation of the correlation between SKP1 and YAP target genes. **b** The expression of SKP1 positively correlates with YAP target genes as analyzed by spearman analysis with RNA isolated from 30 cancer tissues of colorectal cancer patients. The total RNA was extracted from clinical samples. The mRNA levels of indicated genes were analyzed by qRT-PCR. Spearman analysis was employed for investigation of the correlation between SKP1 and YAP target genes. **c** The mRNA levels of YAP target genes were upregulated by SKP1 overexpression in colorectal cancer cells. The mRNA of YAP target genes in indicated cells were analyzed by qRT-PCR. **d** The phosphorylation levels of YAP in indicated cells were analyzed by western blot. **e** The protein level of nuclear YAP in indicated cells was analyzed by western blot. **f** SKP1 promotes nuclear translocation of YAP in colorectal cancer cells. The localization of YAP in indicated cells was analyzed by immunostaining. Student’s *t*-test (***p < 0.001)

To investigate the underlying mechanism of SKP1 promoting CRC stemness, we screened the correlated genes of SKP1 in TCGA dataset by Spearman correlation analysis. As shown in Fig. 4a, we found that the expression of SKP1 is positively correlated with YAP target genes. This result was further confirmed by fresh CRC samples (Fig. 4b). In addition, the upregulated mRNA levels of YAP target genes were observed in SKP1-overexpressing HCT-116 and #1 primary cells (Fig. 4c). These results indicated the stimulatory role of SKP1 in regulation of Hippo/YAP signaling. We next found that the phosphorylation level of YAP was downregulated in SKP1-overexpressing cells (Fig. 4d). Furthermore, the protein level of the nuclear YAP was significantly upregulated by SKP1 overexpression (Fig. 4e). This result was further confirmed by immunostaining (Fig. 4f). The opposite results were obtained in SKP1-knockdown CRC cells (Additional file 2: Figure S3A, 3b, c). Taken together, these results demonstrated that SKP1 activates Hippo/YAP signaling pathway in CRC cells.

### YAP activation is necessary for SKP1 promoting colorectal cancer stemness

**Fig. 5:**
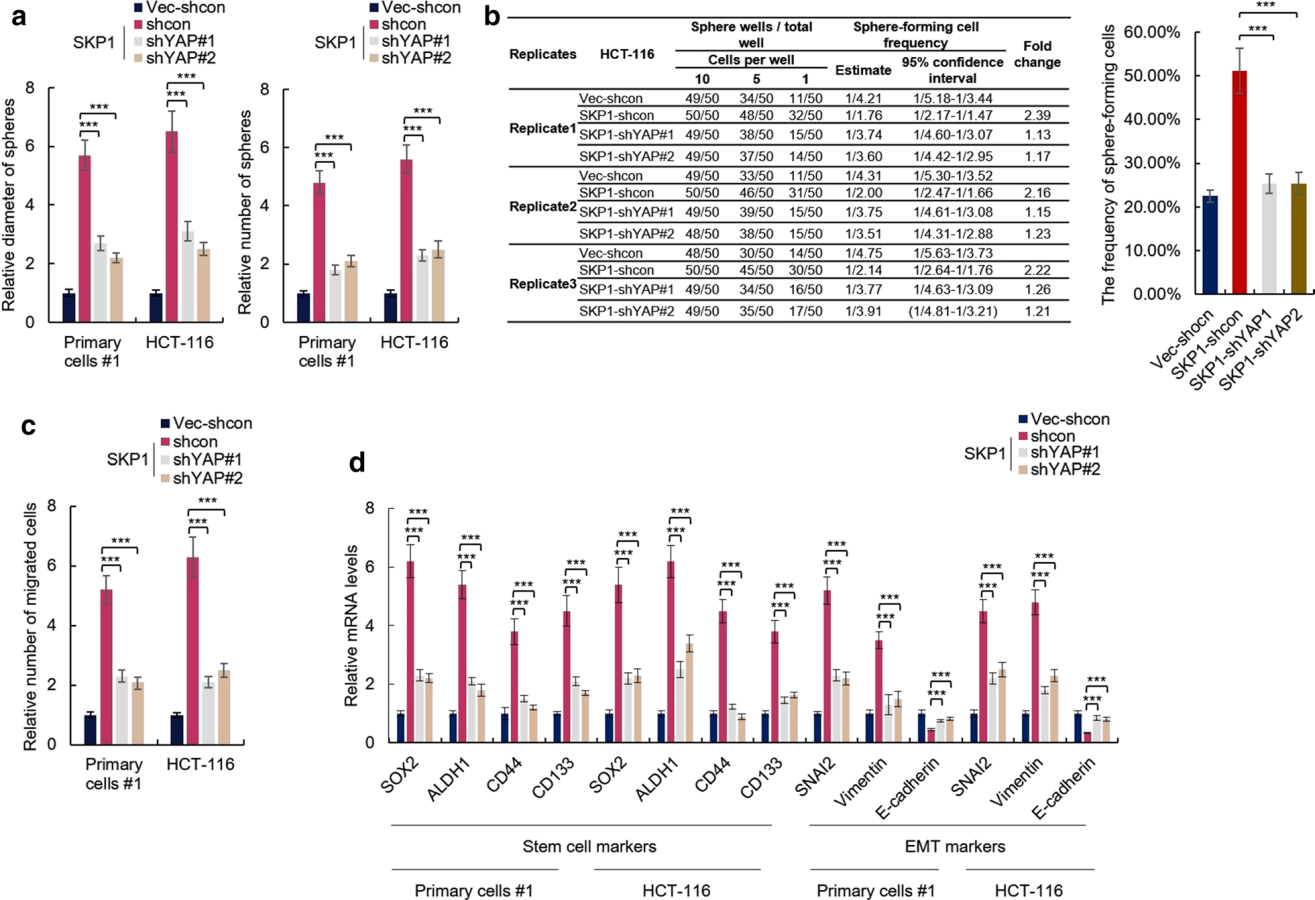
Knockdown of YAP abolished the effect of SKP1 on the stemness of colorectal cancer cells. **a**–**d** Knockdown of YAP abolished the effect of SKP1 on the stemness of colorectal cancer cells. The sphere-forming capacities (**a**), the frequency of sphere-forming cells (**b**), the migration capacity (**c**), the expression of cancer stem cells and EMT markers (**d**) in indicated cells were analyzed by sphere formation (**a**), limiting dilution assay (**b**), transwell (**c**) and qRT-PCR assay (**d**), respectively. Student’s *t*-test (***p < 0.001)

To confirm the necessary role of Hippo/YAP signaling in SKP1 promoting CRC stemness, we depleted YAP in SKP1-overexpressing colorectal cancer cells (Additional file 2: Figure S4). As expected, depletion of YAP significantly abolished the stimulatory effect of SKP1 on sphere-forming capacity (Fig. 5a), the frequency of sphere-forming cells (Fig. 5b) and migration activities (Fig. 5c). Furthermore, depletion of YAP abolished the regulatory effect of SKP1 on the expression of CRC-SCs and EMT markers in CRC cells (Fig. 5d), which confirmed the necessary role of YAP activation in SKP1 promoting CRC stemness.

### SKP1 inhibits RASSF1 at the transcriptional level

**Fig. 6:**
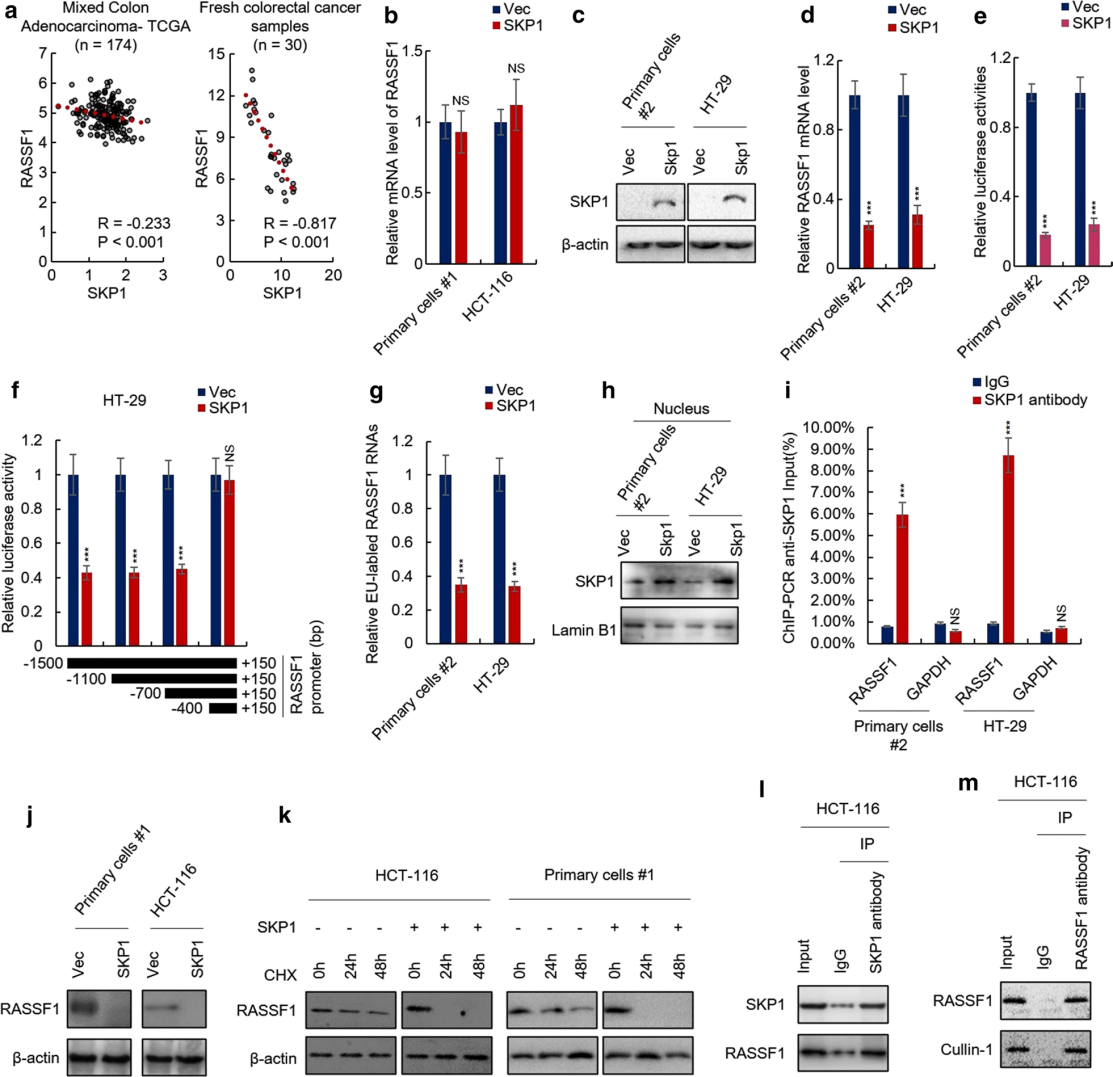
SKP1 negatively regulates RASSF1 at both mRNA and protein level. **a** The correlation between SKP1 and RASSF1 was analyzed by TCGA dataset (left) and clinical samples (right). **b** The mRNA levels of RASSF1 in indicated cells were analyzed by qRT-PCR. **c** Characterization of SKP1-overexpressing HT-29 and #2 primary cells by western blot. **d** The mRNA levels of RASSF1 in indicated cells were analzyed by qRT-PCR. **e**, **f** The transcriptional activity of the promoter of RASSF1 (**e**) and different fraction of RASSF1 promoter (**f**) in indicated cells were analyzed by luciferase reporter assay. **g** The transcription of RASSF1 in indicated cells was analyzed by nuclear run-on assay. **h** The nuclear SKP1 in indicated cells was analyzed by western blot assay. **i** The binding between SKP1 and RASSF1 promoter was analyzed by ChIP-PCR assay. **j**, **k** The RASSF1 protein levels in indicated cells were analyzed by western blot assay. **l**, **m** The binding between SKP1 and RASSF1 (**l**), RASSF1 and Cullin-1 (**m**) were analyzed by immunoprecipitation assay. Student’s *t*-test (***p < 0.001)

To further investigate the mechanism underlying SKP1 activating Hippo/YAP signaling pathway, we screened the correlated genes of SKP1 in Hippo/YAP signaling pathway with the TCGA dataset by Spearman correlation analysis. As shown in Fig. 6a left, RASSF1 is negatively correlated with SKP1, and this result was subsequently confirmed by fresh CRC samples (Fig. 6a, right). However, the downregulation of the mRNA level of RASSF1 was not observed in SKP1-overexpressing HCT-116 and #1 primary cells (Fig. 6b), this is in conflict with Spearman correlation analysis results. We first screened CRC cell lines and primary cells and found that the downregulation of RASSF1 mRNA level was observed in SKP1-overexpressing HT-29 and primary cell #2 (Fig. 6c, d). To confirm this result, we performed luciferase reporter assay and found that SKP1 overexpression significantly inhibited the transcriptional activity of RASSF1 promoter (Fig. 6e), and that − 700 to − 400 bp relative to the transcriptional start site (+ 1) is necessary for this process (Fig. 6f). Moreover, by nuclear run-on assay, the significant reduction of RASSF1 transcription was observed in SKP1-overexpressing HT-29 and #2 primary cells (Fig. 6g). As the roles of SKP1 in nucleus were rarely studied, we next examined whether SKP1 was able to translocate to nucleus and binds the promoter of RASSF1 directly. As shown in Fig. 6h, western blot analysis showed that SKP1 translocated to nucleus and the protein level of nuclear SKP1 was upregulated in SKP1-overexpressing HT-29 and #2 primary cells. Furthermore, ChIP-PCR assay showed that SKP1 directly binds to RASSF1 promoter (Fig. 6i). These results demonstrated that SKP1 serves as a transcription factor or partner that inhibits the transcription of RASSF1.

### SKP1 degrades RASSF1 by directly binding to RASSF1

As we have previously observed the inhibitory effect of SKP1 on Hippo/YAP signaling in HCT-116 and #1 primary cells, we wonder whether RASSF1 is the target of SKP1 in these cells. Through western blot assay, we found that SKP1 inhibits the protein level of RASSF1 in HCT-116 and #1 primary cells (Fig. 6j). Next, we studied the effect of SKP1 on the stabilization of RASSF1. The translation was inhibited by CHX (Cycloheximide) in SKP1-overexpressing HCT-116 and #1 primary cells and the protein levels of RASSF1 in CHX-treated cells were examined. As shown in Fig. 6k, the stabilization of RASSF1 was significantly inhibited by SKP1 in HCT-116 and #1 primary cells. As SKP1 usually play a role as a component of SCF complex, we next performed the co-immunoprecipitation assay to study whether SKP1 directly binds to RASSF1 as well as the component of SCF complex. As shown in Fig. 6l, m, the results from co-immunoprecipitation assays indicated the direct bindings between SKP1 and RASSF1 as well as RASSF1 and Cullin-1, which demonstrated that SKP1 serves as a component of SCF complex to degrade RASSF1.

### Overexpression of RASSF1 abolished the effect of SKP1 on Hippo/YAP signaling and stemness CRC cells

**Fig. 7:**
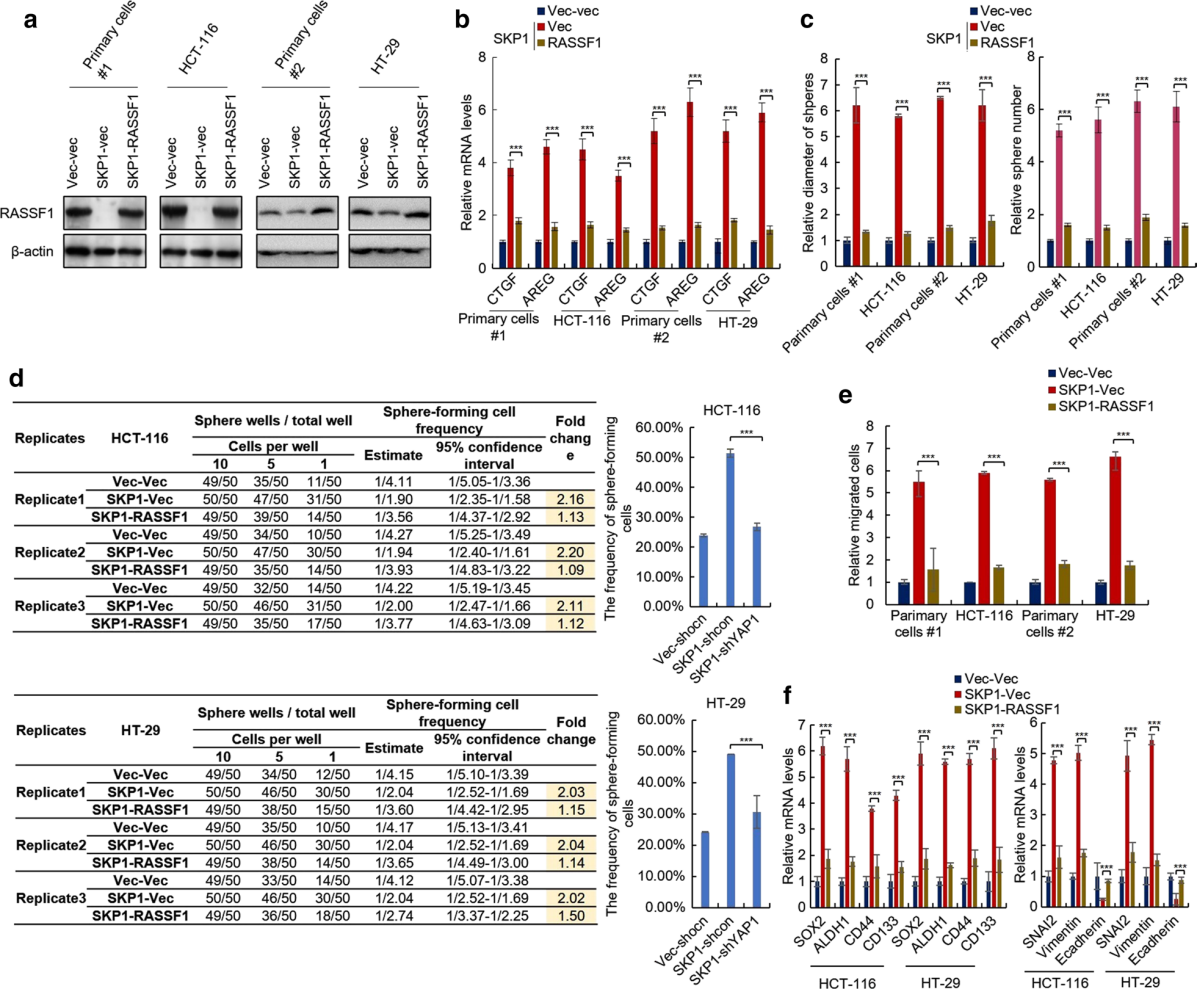
Overexpression of RASSF1 abolished the effect of SKP1 on Hippo/YAP signaling, stemness and EMT of colorectal cancer cells. **a** Characterization of SKP1-overexpressing RASSF-overexpressing colorectal cancer cells. **b** The mRNA levels of YAP target genes in indicated cells were analyzed by qRT-PCR. **c**–**f** The sphere-forming capacities (**c**), frequency of sphere-forming cells (**d**) and migration capacity (**e**) in indicated cells were analyzed by sphere formation (**c**), limiting dilution (**d**) and transwell (**e**) assay, respectively. **f** The mRNA levels of cancer stem cell markers (left) and EMT markers (right) in indicated cells were analzyed by qRT-PCR. Student’s *t*-test (***p < 0.001)

Next, to demonstrate the role of RASSF1 inhibition in the effect of SKP1 on Hippo/YAP signaling and CRC stemness, the RASSF1 was overexpressed in SKP1-overexpressing cells (Fig. 7a). We found that overexpression of RASSF1 abolished the effect of SKP1 on the expression of YAP target genes (CTGF and AREG) (Fig. 7b), sphere-forming capacity (Fig. 7c), the frequency of sphere-forming cells (Fig. 7d), migration capacity (Fig. 7e), and the expression of cancer stem cell markers (SOX2, ALDH1, CD44 and CD133) (Fig. 7f, left) and EMT markers (SNAI2, Vimentin and E-cadherin) (Fig. 7f, right) in CRC cells, which confirmed that RASSF1 is the target of SKP1 inhibiting Hippo/YAP signaling and CRC stemness. Taken together, these results demonstrated that SKP1 promotes YAP-mediated colorectal cancer stemness via suppressing RASSF1 at both mRNA and protein levels.

## Discussion

In this study, we found that SKP1 inhibits RASSF1 at both mRNA and protein level, activates Hippo/YAP signaling pathway, and thereby promoting the stemness of CRC-SCs. This finding revealed a novel mechanism underlying the maintenance of the stemness of CRC-SCs and identified a novel drug target for targeting CRC-SCs.

Colorectal cancer is a predominant cancer and accounts for approximately ten percent of cancer-related mortality [1–3]. In spite of emerging new treatments, few changes in long-term survival rate can be detected. The failure of a complete cure may be the result of the lack of complete eradication of CRC-SCs [7, 8]. CSCs possess stem cell-like properties and contribute to cancer initiation, progression, metastasis and recurrence as well as therapeutic resistance, making them attractive as drug targets [9, 10]. Therefore, it is very important to develop strategies for targeting and eradicating CRC-SCs.

Identifying novel mechanisms underlying the maintenance of the stemness of CRC-SCs is one of the most important work for developing novel therapeutic strategies. Although it was previously reported that SCF complexes plays a crucial role in cancer development, its effect in CRC-SCs is not fully understood. In this study, we found that overexpression of SKP1 promotes the stemness of CRC-SCs and knockdown of SKP1 leads to the opposite results. These results support the stimulatory role of SKP1 in the maintenance of the stemness of CRC-SCs and thus revealed that SKP1 is an important player for CRC-SCs maintenance.

Another finding in this study is that Hippo/YAP signaling pathway is the main downstream effector of SKP1 and SCF complex. Although previous studies have reported that SCF complex is involved in YAP degradation [27], the roles of SKP1 and SCF complex in regulation of Hippo/YAP signaling are incompletely understood. We found that SKP1 positively regulates YAP activity and knockdown of YAP or overexpression of YAP negative regulator, RASSF1, significantly abolished the effect of SKP1 on CRC-SCs stemness. This result demonstrated that the key component, RASSF1, in Hippo signaling is the prime target of SKP1 that mediates the stimulatory effect of SKP1 on YAP activity as well as colorectal cancer stemness, which indicated that both SKP1 and YAP inhibitor could be effective strategies for eradicating CRC-SCs. Furthermore, as an adaptor protein in SCF complex, SKP1 possesses a broad function in the cells, especially in normal cells; as the result, inhibition of the main downstream pathways, Hippo/YAP signaling, would be a feasible strategy to reduce the side-effect of anti-cancer treatment targeting SKP1.

Our results also suggest a novel mechanism underlying SKP1 action. SKP1 is known as a component of SCF (SKP1/Cullin-1/F-box) complexes, which regulates the ubiquitination of specific protein substrates for degradation by the proteasome. However, few is known about its role as transcription factor or partner. In this study, we found that SKP1 not only degrades RASSF1 by form SCF complex, but also directly binds to the promoter of RASSF1 and inhibits RASSF1 transcription, which suggests that SKP1 also functions as a transcription factor or partner.

## Conclusion

In conclusion, our results demonstrated that SKP1 promotes YAP-mediated colorectal cancer stemness via degradation of RASSF1.

## Supplementary information

**Additional file 1. sheet 1**: Gene expression data of related genes in TCGA dataset;** sheet 2**: Gene expresison data of related genes in 30 colorectal cancer samples.

**Additional file 2. Figure S1**: Characterization of SKP1-overexpressing CRC cells;** Figure S2**. Characterization of SKP1-knockdown CRC cells;** Figure S3**: SKP1 knockdown inhibits the Hippo/YAP signaling in colorectal cancer cells. (A) The mRNA levels of YAP target genes were downregulated by SKP1 knockdown in colorectal cancer cells. The mRNA levels of YAP target genes in indicated cells were analyzed by qRT-PCR. (B) The phosphorylation level of YAP in indicated cells was analyzed by western blot. (C) The protein level of nuclear YAP in indicated cells was analyzed by western blot. Student’s t-test (***p < 0.001);** Figure S4**. Characterization of YAP-knockdown SKP1-overexpressing CRC cells.

## Data Availability

All data and materials are available from the corresponding authors.

## Abbreviations

CRC: Colorectal cancer
CSCs: Cancer stem cells
SKP1: S-Phase Kinase Associated Protein 1
SCF: SKP1/Cullin-1/F-box
CRC-SCs: Colorectal cancer stem cells
FCS: Fetal calf serum
