# Supplementary material for: SKP1 promotes YAP-mediated colorectal cancer stemness via suppressing RASSF1
Source: Cancer Cell Int. 2020 Dec 3;20:579. doi: 10.1186/s12935-020-01683-0 (PMC7713163; doi:10.1186/s12935-020-01683-0)
Supplement: Supplementary file 2 — Additional file 2. Figure S1: Characterization of SKP1-overexpressing CRC cells; Figure S2. Characterization of SKP1-knockdown CRC cells; Figure S3: SKP1 knockdown inhibits the Hippo/YAP signaling in colorectal cancer cells. (A) The mRNA levels of YAP target genes were downregulated by SKP1 knockdown in colorectal cancer cells. The mRNA levels of YAP target genes in indicated cells were analyzed by qRT-PCR. (B) The phosphorylation level of YAP in indicated cells was analyzed by western blot. (C) The protein level of nuclear YAP in indicated cells was analyzed by western blot. Student’s t-test (***p < 0.001); Figure S4. Characterization of YAP-knockdown SKP1-overexpressing CRC cells. [file 12935_2020_1683_MOESM2_ESM.docx]

Running title: SKP1 promotes colorectal cancer stemness

**SKP1 promotes YAP-mediated colorectal cancer stemness via suppressing RASSF1**

Cong Tian^1,2*^, Tingyuan Lang^3*^, Jiangfeng Qiu^4*^, Kun Han^1,2^, Lei Zhou^5,6,7^, Daliu Min^1,2^, Zhiqi Zhang^8,9#^, Dachuan Qi^9#^

^1^Department of Medical Oncology, Shanghai University of Medicine & Health Sciences Affiliated Sixth People’s Hospital East Campus, No. 222 HuanhuXisan Road,Pudong New Area,Shanghai, 201306, P.R. China

^2^Department of Medical Oncology, Shanghai Jiao Tong University Affiliated Sixth People's Hospital East Campus, No. 222 Huan Hu Xi San Road, Pudong New Area, Shanghai 201306, P.R. China.

^3^Department of Gynecologic Oncology, Chongqing University Cancer Hospital & Chongqing Cancer Institute & Chongqing Cancer Hospital, Chongqing 400030, Chongqing, P.R. China.

^4^Department of Gastrointestinal Surgery, Renji Hospital Shanghai Jiao Tong University School of Medicine, Shanghai 200127, P.R. China.

^5^Singapore Eye Research Institute, The academia, 20 College Road, Discovery Tower Level 6, Singapore, 169856, Singapore.

^6^Department of Ophthalmology, Yong Loo Lin School of Medicine, National University of Singapore, 1E Kent Ridge Road, NUHS Tower Block Level 7, Singapore, 119228, Singapore.

^7^Ophthalmology and Visual Sciences Academic Clinical Research Program, Duke-NUS Medical School, 8 college Road, Singapore, 169867, Singapore.

^8^Department of General Surgery, Shanghai University of Medicine & Health Sciences Affiliated Sixth People’s Hospital East Campus, No. 222 HuanhuXisan Road,Pudong New Area,Shanghai, 201306, P.R. China

^9^Department of General Surgery, Shanghai Jiao Tong University Affiliated Sixth People’s Hospital, No. 600 Yishan Road, Xuhui District, Shanghai, 200233, P.R. China.

*These authors contributed equally to this work.

**Correspondence:**

**Zhiqi Zhang**

Department of Medical Oncology, Shanghai University of Medicine & Health Sciences Affiliated Sixth People’s Hospital East Campus, No. 222 HuanhuXisan Road,Pudong New Area,Shanghai, 201306, P.R. China**.**

Department of Medical Oncology, Shanghai Jiao Tong University Affiliated Sixth People's Hospital East Campus, No. 222 Huan Hu Xi San Road, Pudong New Area, Shanghai 201306, P.R. China.

Tel +86 189 6435 5142 Fax +86 21 3829 7000 Email: [zzq72@163.com](mailto:zzq72@163.com)

**Dachuan Qi**

Department of General Surgery, Shanghai Jiao Tong University Affiliated Sixth People’s Hospital, No. 600 Yishan Road, Xuhui District, Shanghai, 200233, P.R. China. Tel +86 189 3017 2935 Fax +86 21 3829 7000 Email: alexqi7@163.com

**Method details:**

**Primers:**

**For cloning:**

SKP1:

F: 5’- TGATGACCCAGTTCTCTACCA-3’

R: 5’- TCTCCTTCATCATCCATTCCC-3’

RASSF1:

F: 5’- CACCTGCATGTGCTGTCACG-3’

R: 5’- TAAAAGGAAGTGCGGCGCC-3’

**For qRT-PCR:**

SKP1:

F: 5’- GACCATGTTGGAAGATTTGGGA-3’

R: 5’- TGCACCACTGAATGACCTTTT-3’

SOX2:

F: 5’- TACAGCATGTCCTACTCGCAG-3’;

R: 5’- GAGGAAGAGGTAACCACAGGG-3’

ALDH1:

F: 5’-CCGTGGCGTACTATGGATGC-3’;

R: 5’-GCAGCAGACGATCTCTTTCGAT-3’

SNAI2:

F: 5’- CGAACTGGACACACATACAGTG-3’;

R: 5’- CTGAGGATCTCTGGTTGTGGT-3’

CDH1:

F: 5’- ATTTTTCCCTCGACACCCGAT-3’;

R: 5’- TCCCAGGCGTAGACCAAGA-3’

VIM:

F: 5’- TGCCGTTGAAGCTGCTAACTA -3’;

R: 5’- CCAGAGGGAGTGAATCCAGATTA -3’

FGF1:

F: 5’- GCCCTGACCGAGAAGTTTAATC-3’

R: 5’- CCCCGTTGCTACAGTAGAGG-3’

ID2:

F: 5’- GCTATACAACATGAACGACTGCT-3’

R: 5’- AATAGTGGGATGCGAGTCCAG-3’

BIRC2:

F: 5’- AGCACGATCTTGTCAGATTGG-3’

R: 5’- GGCGGGGAAAGTTGAATATGTA-3’

BIRC5:

F: 5’- AGGACCACCGCATCTCTACAT-3’

R: 5’- AAGTCTGGCTCGTTCTCAGTG-3’

RASSF1:

F: 5’- AGGACGGTTCTTACACAGGCT-3’

R: 5’- TGGGCAGGTAAAAGGAAGTGC-3’

GAPDH:

F: 5’-ACAACTTTGGTATCGTGGAAGG-3’;

R: 5’-GCCATCACGCCACAGTTTC-3’

**Antibodies:**

Anti-SKP1 (CST, # 2156, 1:1000); Anti-SOX2 (CST, # 3579, 1:1000); Anti-ALDH1 (CST, # 36671, 1:1000); Anti-E-cadherin (CST, # 3195, 1:1000); Anti-Vimentin (CST, # 5741, 1:1000); Anti-SNAI2 (CST, # 9585, 1:1000); Anti-ID2 (CST, # 3431, 1:1000); Anti-FGF1 (CST, # 3139, 1:1000); Anti-BIRC2 (CST, # 7065, 1:1000); Anti-BIRC5 (CST, # 56674, 1:1000); Anti-β-actin (CST, # 4970, 1:1000); Anti-rabbit IgG, HRP-linked (CST, # 7074, 1:1000);

**Additional Figures**

**
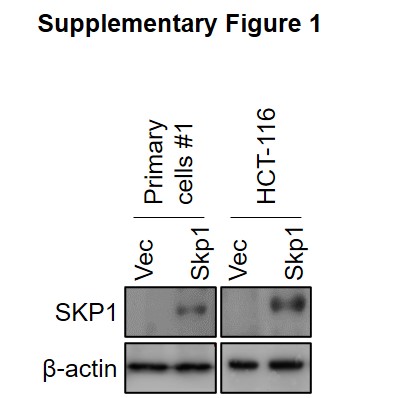
**

**Additional Figure S1. Characterization of SKP1-overexpressing CRC cells.**

The protein levels of SKP1 in indicated cells were detected by western blot.

**
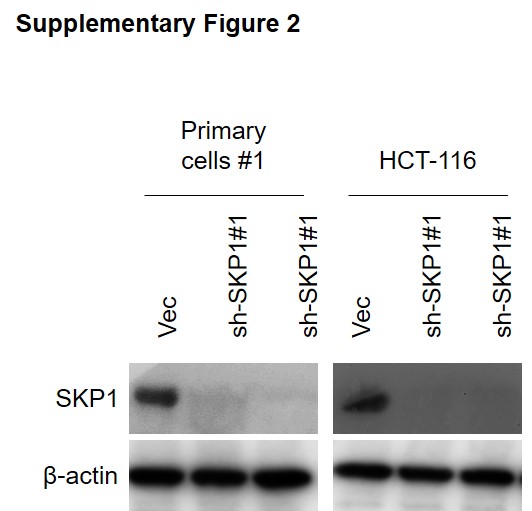
**

**Additional Figure S2. Characterization of SKP1-knockdown CRC cells.**

The protein levels of SKP1 in indicated cells were detected by western blot.

**
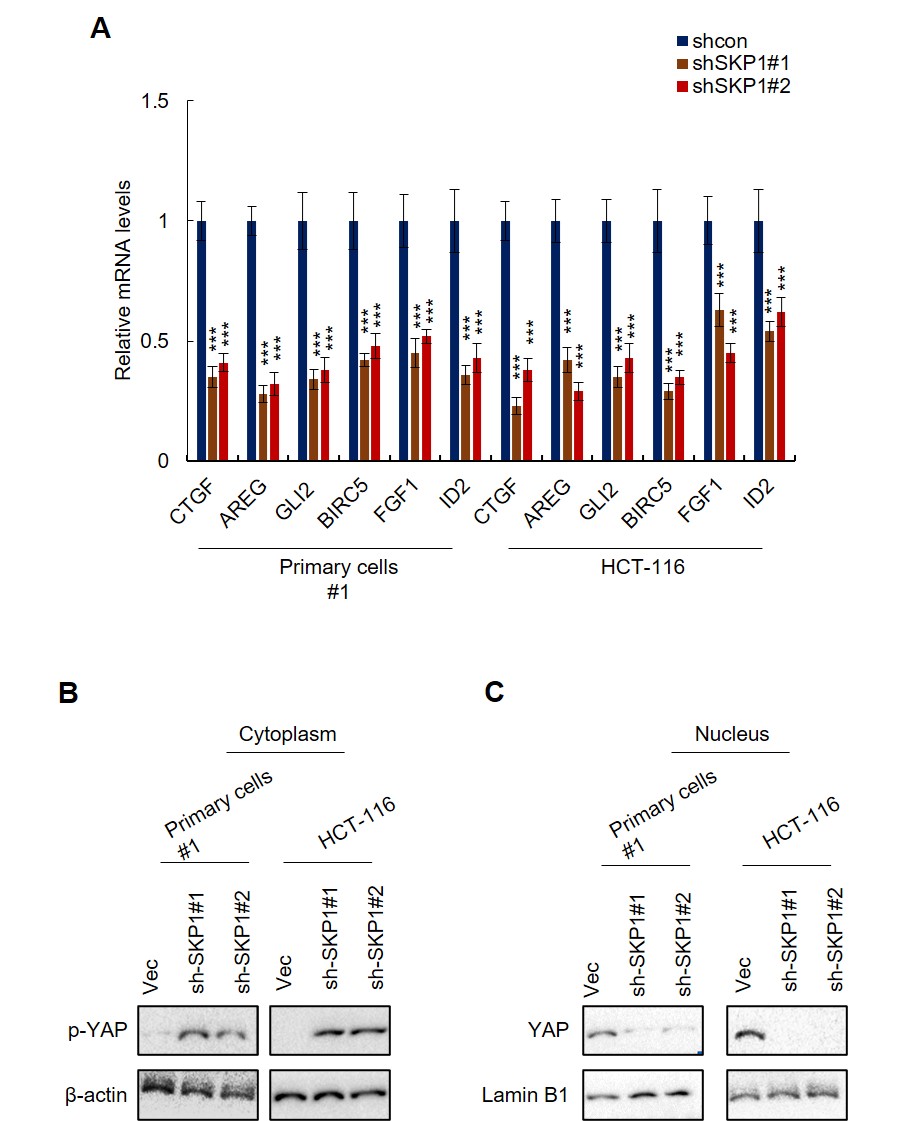
**

**Additional Figure S3. SKP1 knockdown inhibits the Hippo/YAP signaling in colorectal cancer cells.**

**(A)** The mRNA levels of YAP target genes were downregulated by SKP1 knockdown in colorectal cancer cells. The mRNA levels of YAP target genes in indicated cells were analyzed by qRT-PCR. **(B)** The phosphorylation level of YAP in indicated cells was analyzed by western blot. **(C)** The protein level of nuclear YAP in indicated cells was analyzed by western blot. Student’s *t*-test (****p*<0.001).

**
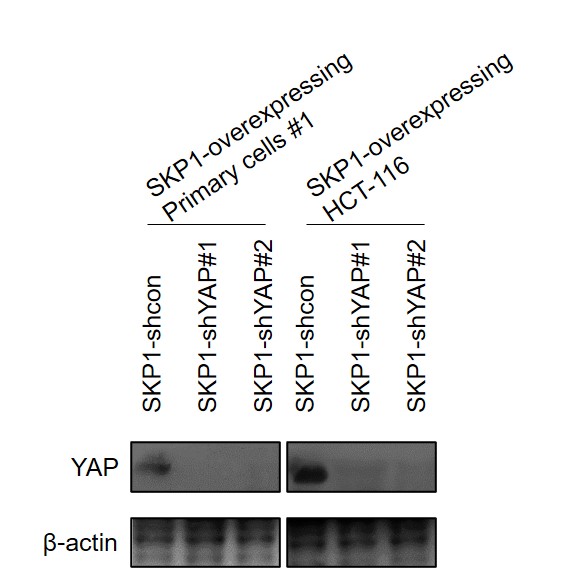
**

**Additional Figure S4. Characterization of YAP-knockdown SKP1-overexpressing CRC cells.** The protein levels of YAP in indicated cells were detected by western blot.
